# Supplementary material for: Mechanisms governing the pioneering and redistribution capabilities of the non-classical pioneer PU.1
Source: Nat Commun. 2020 Jan 21;11:402. doi: 10.1038/s41467-019-13960-2 (PMC6972792; doi:10.1038/s41467-019-13960-2)
Supplement: Supplementary file 7 — Source data [file 41467_2019_13960_MOESM7_ESM.zip › Source_Data/FigureS2/FigureS2B_MotifScanOutput/KmeansCluster13/homerResults/motif15.similar.html]

motif15

## Information for motif15

A
G
T
C
A
T
G
C
C
G
T
A
A
G
T
C
C
G
T
A
A
C
G
T
A
G
T
C
  
Reverse Opposite:  

A
C
T
G
C
G
T
A
A
C
G
T
A
C
T
G
A
C
G
T
A
T
C
G
A
C
T
G
  

|  |  |
| --- | --- |
| p-value: | 1e-28 |
| log p-value: | -6.624e+01 |
| Information Content per bp: | 1.972 |
| Number of Target Sequences with motif | 171.0 |
| Percentage of Target Sequences with motif | 10.11% |
| Number of Background Sequences with motif | 1675.2 |
| Percentage of Background Sequences with motif | 3.71% |
| Average Position of motif in Targets | 103.7 +/- 52.8bp |
| Average Position of motif in Background | 101.2 +/- 65.6bp |
| Strand Bias (log2 ratio + to - strand density) | 0.2 |
| Multiplicity (# of sites on avg that occur together) | 1.03 |
| Motif File: | file (matrix) reverse opposite |

### Similar de novo motifs found

|  |  |  |  |  |  |  |  |
| --- | --- | --- | --- | --- | --- | --- | --- |
| Rank | Match Score | Redundant Motif | P-value | log P-value | % of Targets | % of Background | Motif file |
| 1 | 0.762 | A G T C G A C T A C G T A C G T C T A G G T C A C G A T T A C G C A G T A C T G C A T G A C G T | 1e-25 | -58.880364 | 3.31% | 0.48% | motif file (matrix) |
| 2 | 0.754 | A T G C G A T C T C G A G T A C G T C A A G C T A T G C A G T C C A G T A C T G G A T C A G T C G T A C T A G C | 1e-17 | -40.354505 | 1.06% | 0.03% | motif file (matrix) |
| 3 | 0.770 | A G T C G A C T A T G C A G C T A C G T C A G T A C T G C T G A A C G T A C T G A C G T A C T G A C T G A C G T | 1e-17 | -40.354505 | 1.06% | 0.03% | motif file (matrix) |
